# Effect of Alzheimer's disease on the dynamical and computational characteristics of recurrent neural networks

**DOI:** 10.1186/1471-2202-14-S1-P282

**Published:** 2013-07-08

**Authors:** Claudia Bachmann, Tom Tetzlaff, Susanne Kunkel, Philipp Bamberger, Abigail Morrison

**Affiliations:** 1 Inst. of Neuroscience and Medicine (INM-6) and Inst. for Advanced Simulation (IAS-6), Jülich Research Centre and JARA, Germany; 2 Simulation Laboratory Neuroscience - Bernstein Facility Simulation and Database Technology, Institute for Advanced Simulation, Jülich Aachen Research Alliance, Jülich Research Centre, Germany; 3 Inst. of Cognitive Neuroscience, Faculty of Psychology, Ruhr University Bochum, Germany; 4 Bernstein Center Freiburg, Albert-Ludwigs University, Freiburg, Germany

## 

Recurrent circuits of simple model neurons can provide the substrate for cognitive functions such as perception, memory, association, classification or prediction of dynamical systems [1-3]. In Alzheimer's disease (AD), the impairment of such functions is clearly correlated to synapse loss [4]. So far, the mechanisms underlying this correlation are only poorly understood. Here, we investigate how the loss of excitatory synapses in sparsely connected random networks of spiking excitatory and inhibitory neurons [5] alters their dynamical and computational characteristics. By means of simulations, we study the network response to noisy variations of multidimensional spike-train patterns.

**Figure 1:**
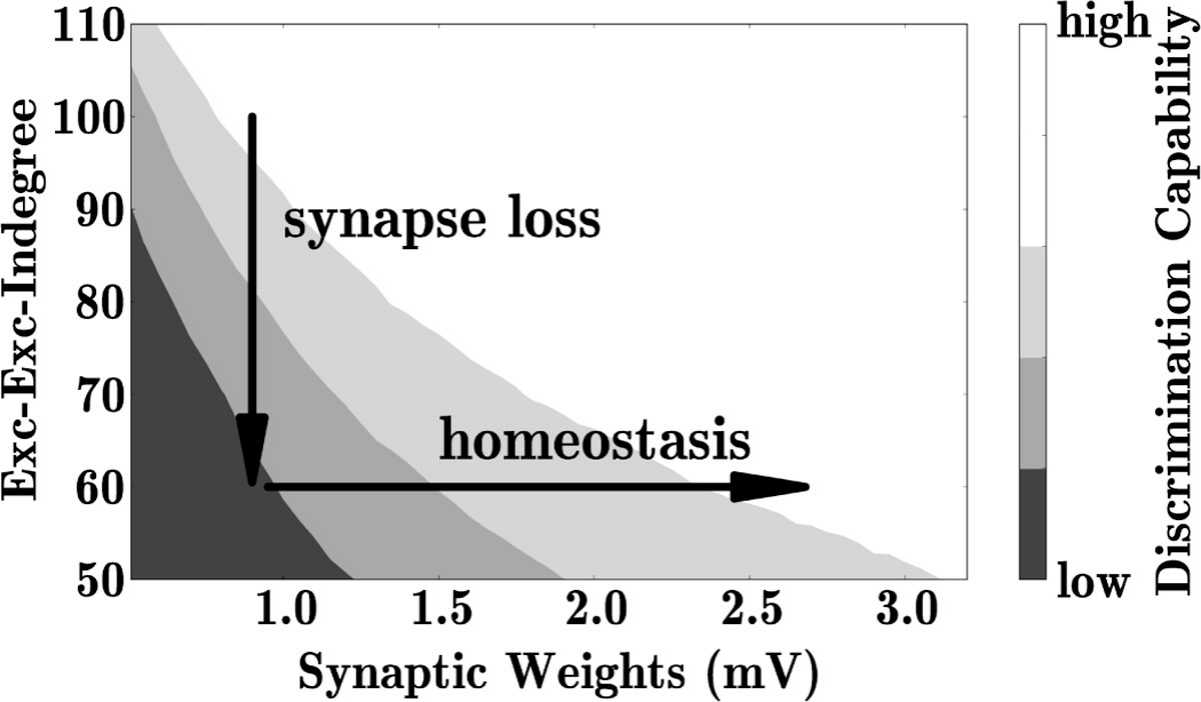
**Loss of excitatory-excitatory synapses (vertical arrow) impairs discrimination capability (gray coded)**. Recovery of discrimination capability by firing-rate homeostasis (scaling up remaining excitatory-excitatory synapses; horizontal arrow).

We find that the loss of excitatory synapses on excitatory neurons (decrease in excitatory-excitatory indegree; vertical arrow in Figure 1) lowers the network's sensitivity to small perturbations of time-varying inputs, reduces its ability to discriminate and improves its generalization capability [6].

A full recovery of the network performance can be achieved by firing-rate homeostasis, implemented by scaling up the remaining excitatory-excitatory synapses (horizontal arrow in Figure 1). Homeostasis may therefore explain the absence of clinical symptoms in early AD, despite cortical damage. The onset of clinical symptoms may result from an exhaustion of homeostatic resources.
